# 1328. Human Parechovirus in Infants: A Case Series

**DOI:** 10.1093/ofid/ofad500.1166

**Published:** 2023-11-27

**Authors:** Tiffany Robles, Roya Gordji, Rachel Downey, Sarmistha Hauger

**Affiliations:** Dell Children's Medical Center; Dell Medical School at the University of Texas at Austin, Austin, Texas; Pediatric Residency at Dell Medical School at the University of Texas at Austin, Austin, Texas; Dell Children's Medical Center of Central Texas, Austin, Texas; Dell Children's Medical Group, Austin, Texas

## Abstract

**Background:**

Human parechovirus (HPeV) is a common virus that causes a myriad of clinical presentations ranging from mild symptoms to severe disease. Signs of severe illness include sepsis, seizures, and meningoencephalitis as well as reports of abnormalities seen on brain imaging associated with neurodevelopmental or cognitive delay. Within the last year, there have been increased reports of severe HPeV in neonates.

**Methods:**

This is a single-center, retrospective case series at a children's hospital in Central Texas. Inclusion criteria were patients from birth to 18 years with cerebrospinal (CSF) biofire positive for HPeV from 4/1/2016 to 2/28/2023. A comprehensive review of all CSF biofire tests in this interval was performed to identify the sample population.

**Results:**

Seventeen children were identified and included in the study. Of these, 13 (76%) presented between 4/1/2022 to 6/30/2022. Laboratory findings included leukopenia in 87% of cases and unremarkable CSF in 94% of cases. Thirteen cases exhibited non-specific fussiness. Three infants had electroencephalogram (EEG) performed, for seizure-like activity or persistent fussiness, of which two had mild encephalopathy. While neither had CSF inflammation, brain magnetic resonance imaging (MRI) revealed areas of restricted diffusion. Repeat brain MRI 4 months from initial revealed resolution of restricted diffusion in both; one had sequelae of moderate volume loss.

**Figure 1:**
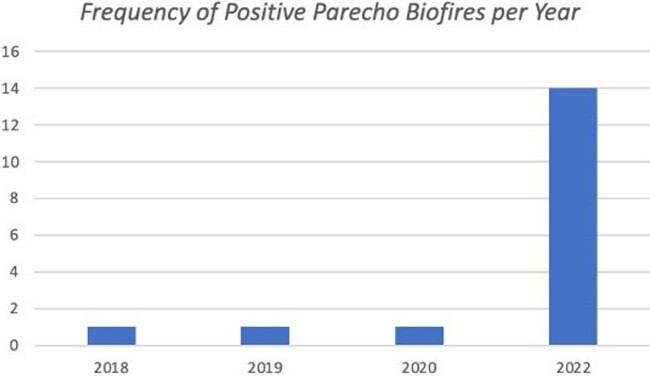
HPeV Frequency

Depicts the number of CSF biofire tests that resulted positive for HPeV by year. There were no cases in 2021 or in 2023 through 2/28/2023. A notable increase in frequency of HPeV is seen in 2022.

**Figure 2:**
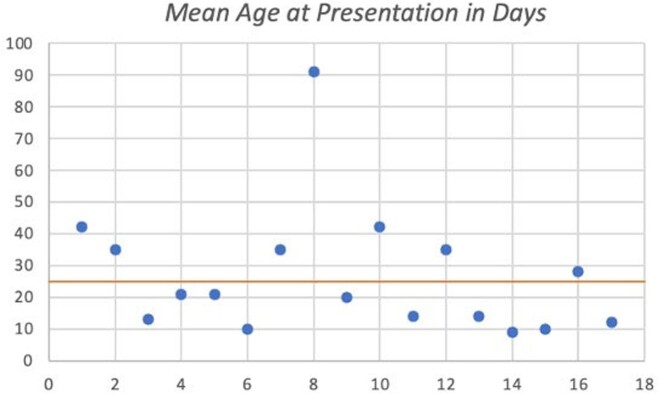
Age at Presentation in Days

Depicts the age at presentation of each patient who tested positive for HPeV in CSF. The mean age at presentation was 26 days.

**Figure 3:**
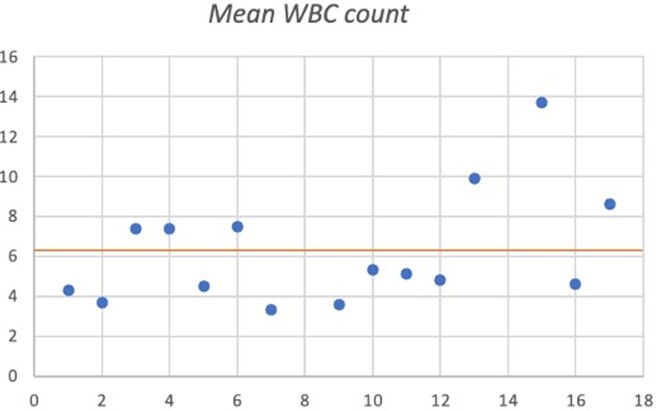
White Blood Count of Patients with CSF Positive HPeV

Depicts the white blood count (WBC) of patients with positive HPeV on CSF biofire. Of note, two cases of positive HPeV were not included given WBC was not collected. The mean WBC was 6.2.

**Conclusion:**

Our data parallel national trends of increased incidence of HPeV identified in 2022, with a cluster of 13 cases within a 3 month period. Cases were primarily neonates. The majority had non-specific fussiness, but there were cases of extensive neurologic involvement. CSF cell counts were relatively unremarkable, even in patients with encephalopathy. Generalized leukopenia was a common finding, thus may be an indicator of HPeV infection. Importantly, of the two infants with encephalopathy and changes on neuroimaging, one had imaging that remained persistently abnormal and clinically had mild delays in gross and fine motor functioning.

**Disclosures:**

**All Authors**: No reported disclosures

